# Minimal Effect of Bevacizumab Treatment on Residual Vestibular Schwannomas after Partial Resection in Young Neurofibromatosis Type 2 Patients

**DOI:** 10.3390/cancers11121862

**Published:** 2019-11-25

**Authors:** Isabel Gugel, Lan Kluwe, Julian Zipfel, Christian Teuber, Marcos Tatagiba, Victor-Felix Mautner, Martin Ulrich Schuhmann, Florian Grimm

**Affiliations:** 1 Department of Neurosurgery, University Hospital Tübingen, BW 72076 Tübingen, Germany; julian.zipfel@med.uni-tuebingen.de (J.Z.); teuber_christian@web.de (C.T.); marcos.tatagiba@med.uni-tuebingen.de (M.T.); martin.schuhmann@med.uni-tuebingen.de (M.U.S.); florian.grimm@med.uni-tuebingen.de (F.G.); 2 Centre of Neurofibromatosis and Rare Diseases, University Hospital Tübingen, BW 72076 Tübingen, Germany; v.mautner@uke.de; 3 Department of Neurology, University Medical Center Hamburg-Eppendorf, HH 20251 Hamburg, Germany; kluwe@uke.de; 4 Department of Maxillofacial Surgery, University Medical Center Hamburg-Eppendorf, HH 20251 Hamburg, Germany; 5 Division of Pediatric Neurosurgery, University Hospital Tübingen, BW 72076 Tübingen, Germany

**Keywords:** bevacizumab, growth rate, neurofibromatosis type 2, vestibular schwannoma

## Abstract

Hearing-preserving partial resection of neurofibromatosis type 2 (NF2) associated vestibular schwannomas (VS) is a preferred treatment strategy, particularly for children and adolescents. However, the residual tumors do grow and lead at some point to continued hearing deterioration. An adjuvant bevacizumab treatment may provide an option for slowing down this process. In this retrospective study, we reviewed tumor volume and hearing data of 16 operated VS in nine patients younger than 30 years over a period of 63 to 142 months. All these patients had one or more bevacizumab treatment periods and most of them had a non-treatment period after surgery. Four different patterns of growth were observed for the residual tumors: (1) growth in the non-treatment periods, which slowed down in the treatment periods; (2) growth slowed down in one but not in another on-period; (3) unaffected growth; (4) no or minimal growth regardless of the treatment. Neither radiological regression of tumor volume nor hearing improvement were observed in the treatment periods. Accelerated hearing deterioration was observed in several non-treatment periods, but also in some treatment periods. No straightforward correlation can be drawn between tumor growth and hearing scores. Tumor growth and worsening of hearing between two measurement points were slightly less in the treatment periods; however, the differences were not significant, because variations were large. In conclusion, our comprehensive follow-up on 16 VS in nine NF2 patients did show heterogenous effects of bevacizumab on small residual vestibular schwannomas after surgery both regarding tumor size and hearing preservation. Thus, smaller and slower growing tumor residuals seem to behave differently to bevacizumab than reported for not-operated faster growing VS.

## 1. Introduction

Neurofibromatosis type 2 (NF2) is an autosomal-dominant tumor predisposition disorder caused by the inactivation of the *NF2* tumor suppressor gene located on chromosome 22q12 and the functional loss of its protein product merlin (moesin-ezrin-radixin-like protein) [1]. The typical hallmark of the disease and key diagnostic criteria are bilateral vestibular schwannomas (VS) and the occurrence of multiple nervous system lesions, including meningiomas, schwannomas and ependymomas. VS-associated comorbidities such as progressive hearing loss, gait disturbances, dizziness and facial palsy are often predominant in childhood and adolescence [2,3]. Genotype–phenotype correlations are known, but the course of the disease is difficult to estimate [4,5]. Radical microsurgery with total tumor removal carries a high risk of hearing loss and facial palsy. By contrast, partial resection aiming at the decompression of the internal auditory canals in young NF2 patients provides a more conservative option with improved growth control and hearing stabilization [6,7]. However, the risk of continuing growth of the residual tumor and hearing deterioration remains. 

An alternative treatment for non-operated VS is a chemotherapy with the vascular endothelial growth factor (VEGF) inhibitor [8]. An anti-VEGF treatment, for example, with bevacizumab, stabilizes tumor growth and sustains or even improves hearing function [9,10]. However, long-term side effects of VEGF inhibitors have to be considered and treatment in childhood remains controversial. It is highly desirable to postpone such chemotherapy as long as possible. 

In this study, 16 progressive VS in nine young NF2 patients were initially partially resected and treated with bevacizumab for various periods at a later timepoint when hearing deteriorated again. Growth of residual tumors and hearing function were followed over a period of 63 to 142 months covering the preoperative period, the postoperative non-treatment period and the postoperative bevacizumab-treatment period. 

## 2. Results

### 2.1. Patients, Tumors and Operation

Clinical, genetic and demographic data and parameters regarding the nine patients are summarized in Table 1 and Table 2. All patients were under the age of 25 years at diagnosis. None of them had a family history of NF2. 

### 2.2. Postoperative Bevacizumab and Toxicity

All patients received bevacizumab with an initial dose of 5 mg per kg every 2 weeks. Over the treatment course and in cases with stabilized tumor growth and hearing, the dose was reduced to minimize long-term toxicity effects. For two patients, no adverse effects of bevacizumab were observed over the entire treatment period. Five other patients had mild adverse effects including fatigue and dryness of the skin and mucous membranes. The last two patients had grade 3 adverse events in the form of proteinuria and arterial hypertension, which necessitated the use of appropriate medication (cases 1 and 17). Bevacizumab treatment had to be transiently discontinued but could be resumed after 3 months, since the adverse effects were manageable with medication. Both patients were treated with angiotensin-converting enzyme inhibitors (ACE inhibitors) and one patient (patient 1) additionally received a beta blocker during the first 6 months after the initiation of medication. Antihypertensive medication in patient 17 was limited to the short treatment-free interval and in patient 1, it was continued until the end of the observation period but in a reduced dosage. We did not see any significant tumor growth and/or hearing change due to the antihypertensive medication. In the other patients, no ancillary medication was used during the bevacizumab treatment. 

### 2.3. Growth of the Residual VS

**Figure 1:**
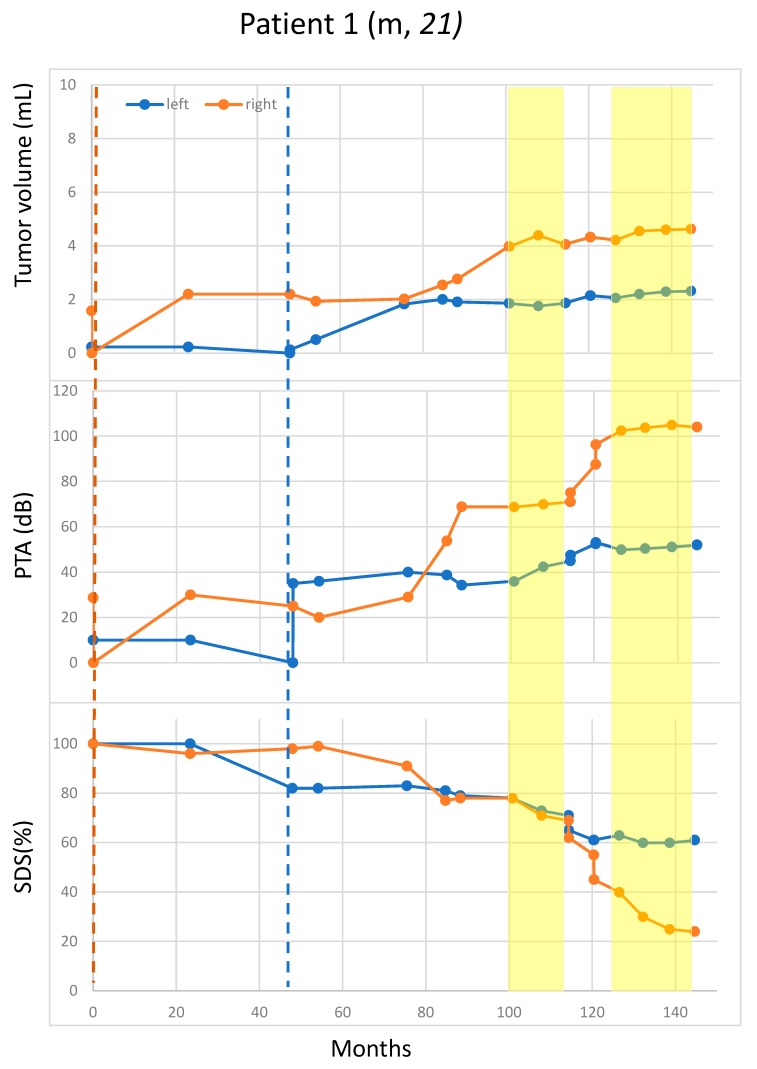
Suppressed growth of residual vestibular schwannoma (VS) in the two bevacizumab-treatment periods. Tumor size was measured in volume (mL). Blue and orange dots represent the measurement for the left and right VS, respectively. Vertical blue and orange lines indicate the surgery times for the left and the right VS, respectively. Bevacizumab-treatment periods are marked in yellow. To enable comparison, same scales were used for all Figure 1, Figure 2, Figure 3 and Figure 4. Patient 1: Tumor growth and hearing function behave differently on both sides at certain time points. Bevacizumab was initiated due to tumor growth and hearing deterioration, especially because of the right-sided tumor, and then slowed down in the treatment period. Overall, the left tumor appeared to be less aggressive in terms of tumor volume acceleration and hearing deterioration at lower baseline volumes. Nevertheless, a more rapid tumor growth could be observed within the first 25 months immediately after the left-sided surgery. Due to arterial hypertension and proteinuria, the medication had to be paused. After drug adjustment for the side-effects, bevacizumab could be restarted. Within drug interruption, there was no accelerated growth but a rapid hearing deterioration was observed. This patient is still under bevacizumab treatment and antihypertensive medication. m; male; PTA: pure-tone average; SDS: speech discrimination score.

The growth of the residual VS in the nine patients after surgery in the treatment and non-treatment periods could be divided into four patterns:

Pattern 1 (one patient): growth in the 1st non-treatment period, stopped/decreased in the following two treatment periods (Figure 1). 

**Figure 2:**
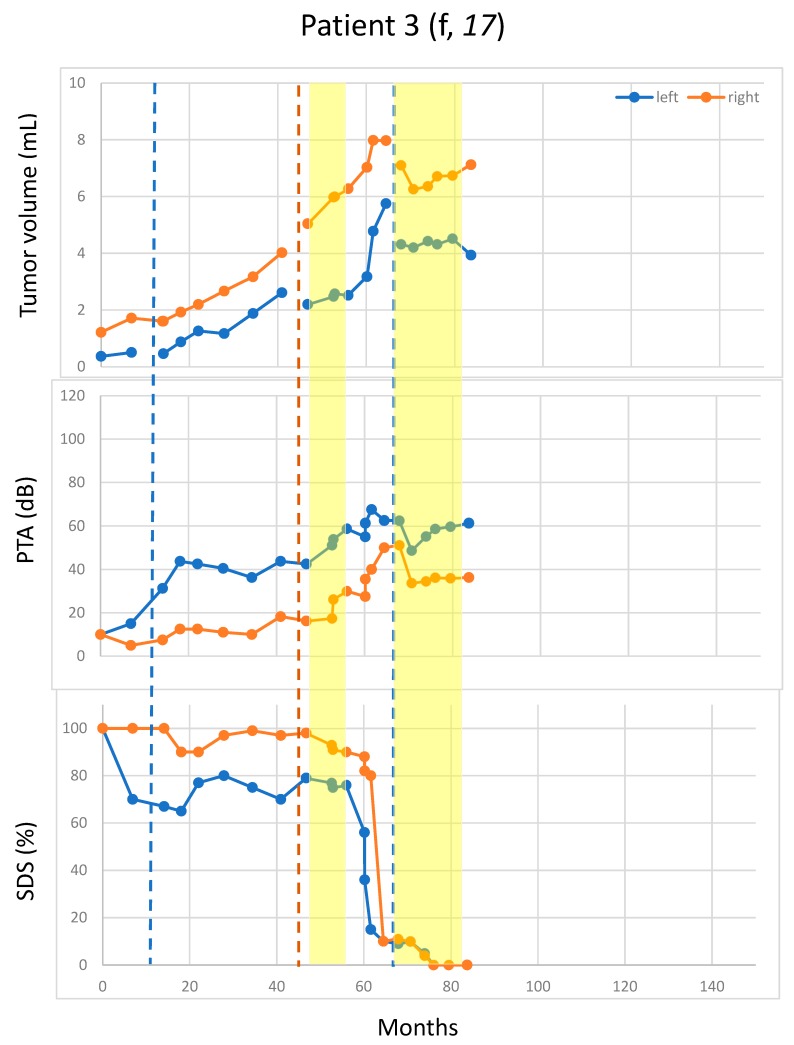

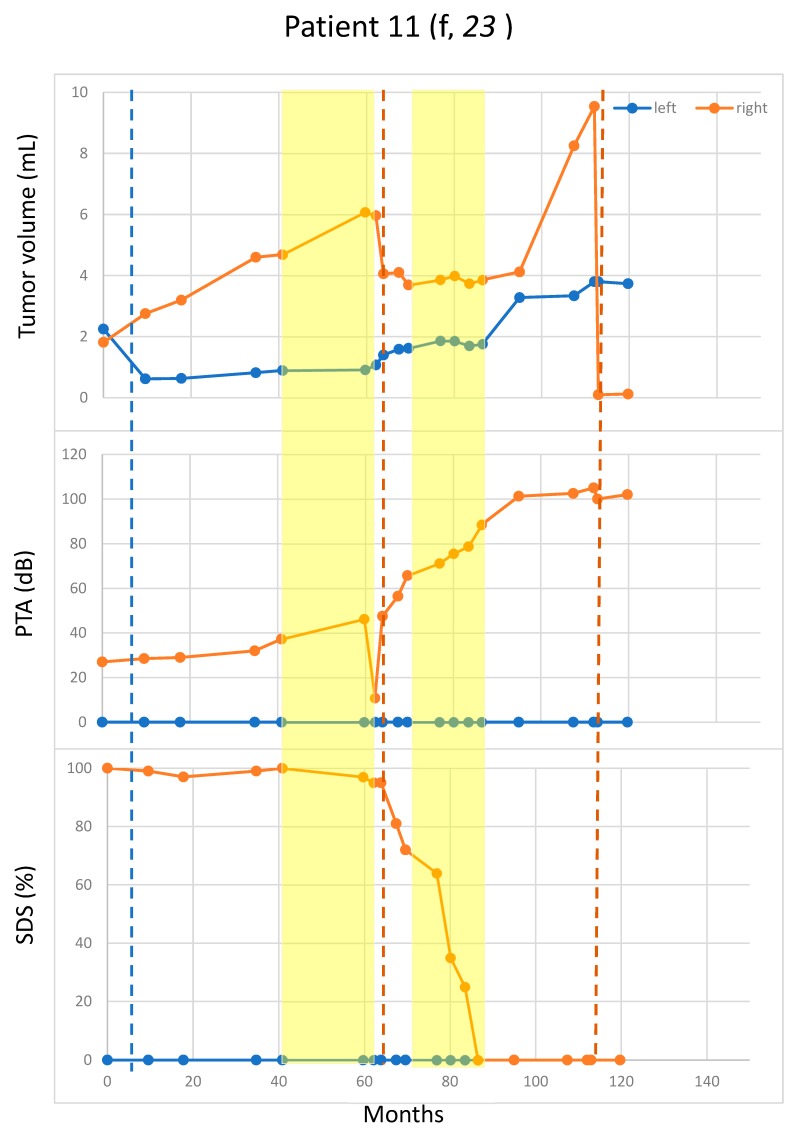
Growth of residual vestibular schwannoma (VS) in one but not in the other bevacizumab-treatment periods. Tumor size was measured in volume (mL). Blue and orange dots represent the measurement for the left and right VS, respectively. Vertical blue and orange lines indicate the surgery times for the left and the right VS, respectively. Bevacizumab-treatment periods are marked in yellow. Patient 3: Rapid tumor growth was observed on both tumor sides after interruption of bevacizumab-treatment in the treatment-free interval. Due to rapid growth and hearing deterioration within a few months, a second surgery was performed on the left tumor on the poorly hearing ear to prevent premature total hearing loss. After the 2nd surgery, bevacizumab was immediately resumed and both tumors responded well. Patient 11: Less aggressive growth dynamics and better hearing function can be seen in the left tumor with better baseline values before treatment. An acceleration of tumor volume was seen on the right side within the first treatment period and, therefore, surgery was performed. Since postoperative right-sided hearing function deteriorated again and further growth of the left tumor was observed, bevacizumab was restarted and a stronger response to treatment was observed for the right tumor, but also for the left tumor. Interestingly, there appears to be growth acceleration on the residual tumor on the left following the first treatment, and similarly, following the second treatment on the right side. Both sides had no growth during the second treatment. Significant reduction of tumor volume after surgery is seen on both sides. f: female; PTA: pure-tone average; SDS: speech discrimination score.

Pattern 2 (two patients): growth in 1st and suppressed growth in the 2nd treatment period. (Figure 2). 

**Figure 3:**
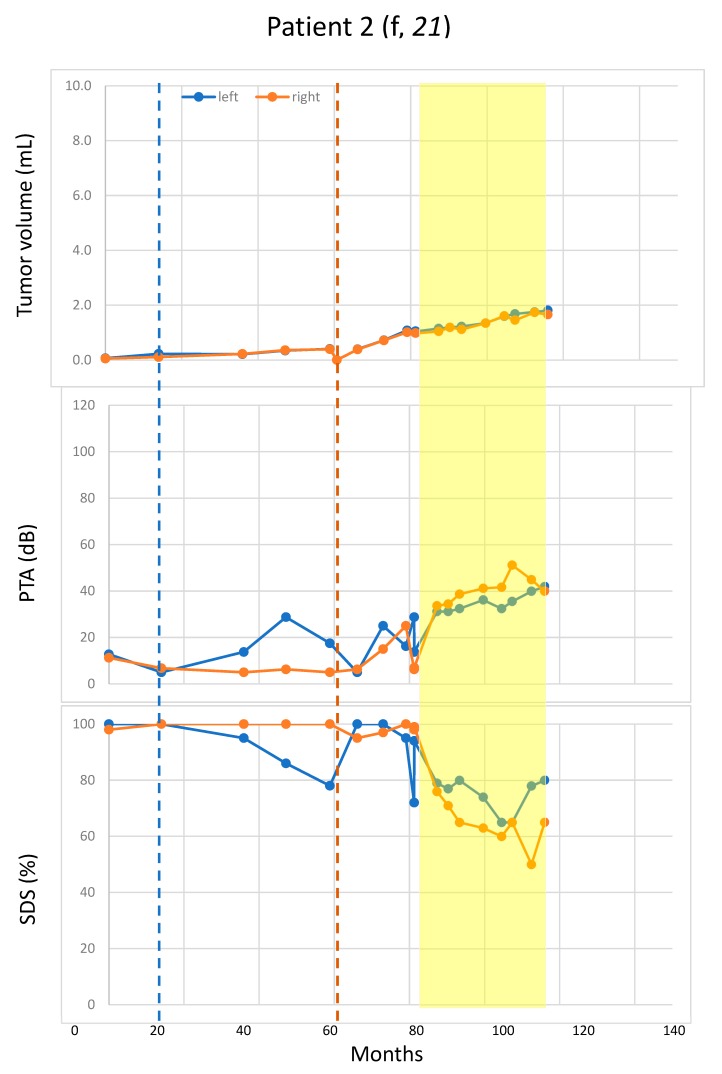

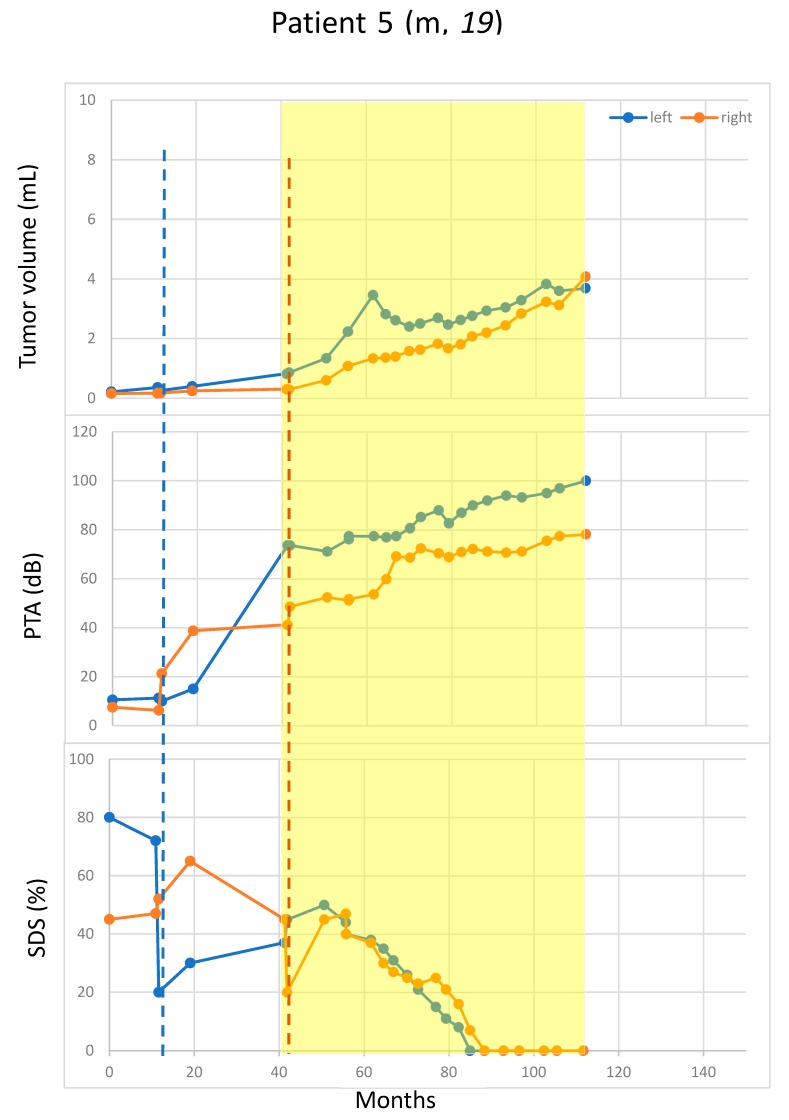
Growth of residual VS in both the treatment and the non-treatment periods. Tumor size was measured in volume (mL). Blue and orange dots represent the measurement for the left and right VS, respectively. Vertical blue and orange lines indicate the surgery times for the left and the right VS, respectively. Bevacizumab-treatment periods are marked in yellow. Patient 2 and Patient 5: No significant tumor volume reduction after surgery could be achieved on either side of both patients due to deterioration of intraoperative brainstem auditory evoked potentials. f: female; m: male; PTA: pure-tone average; SDS: speech discrimination score.

Pattern 3 (two patients): continued growth in both the treatment and the non-treatment periods (Figure 3).

**Figure 4:**
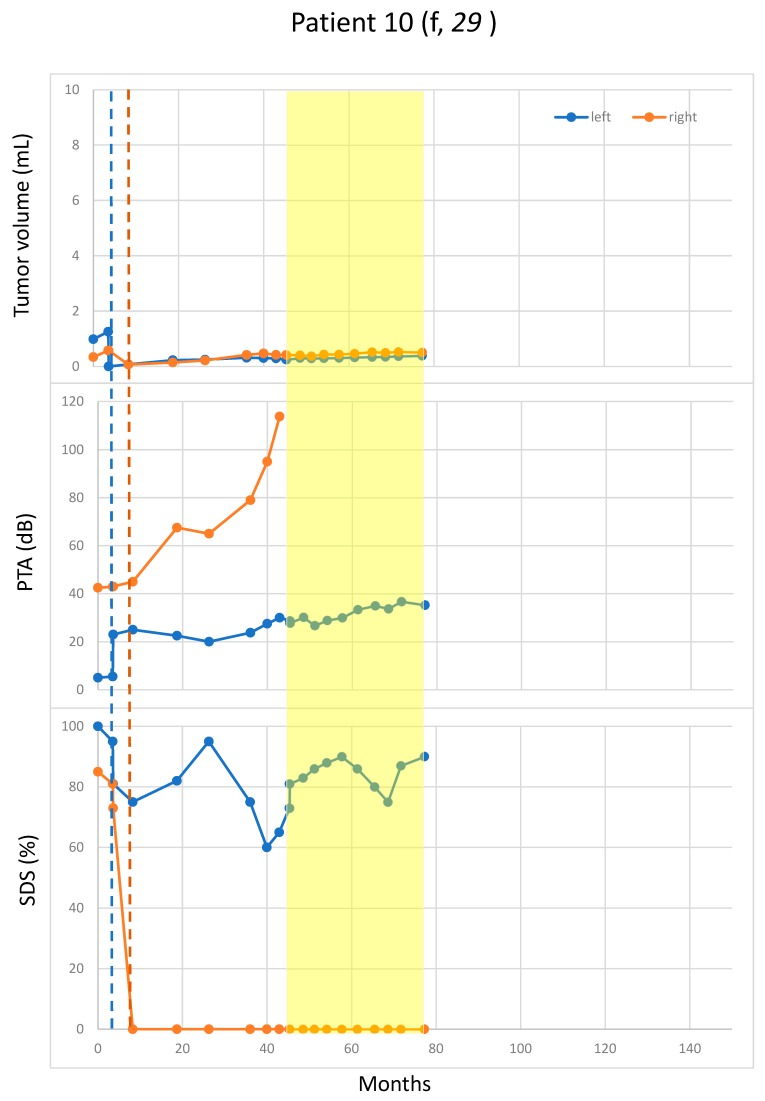

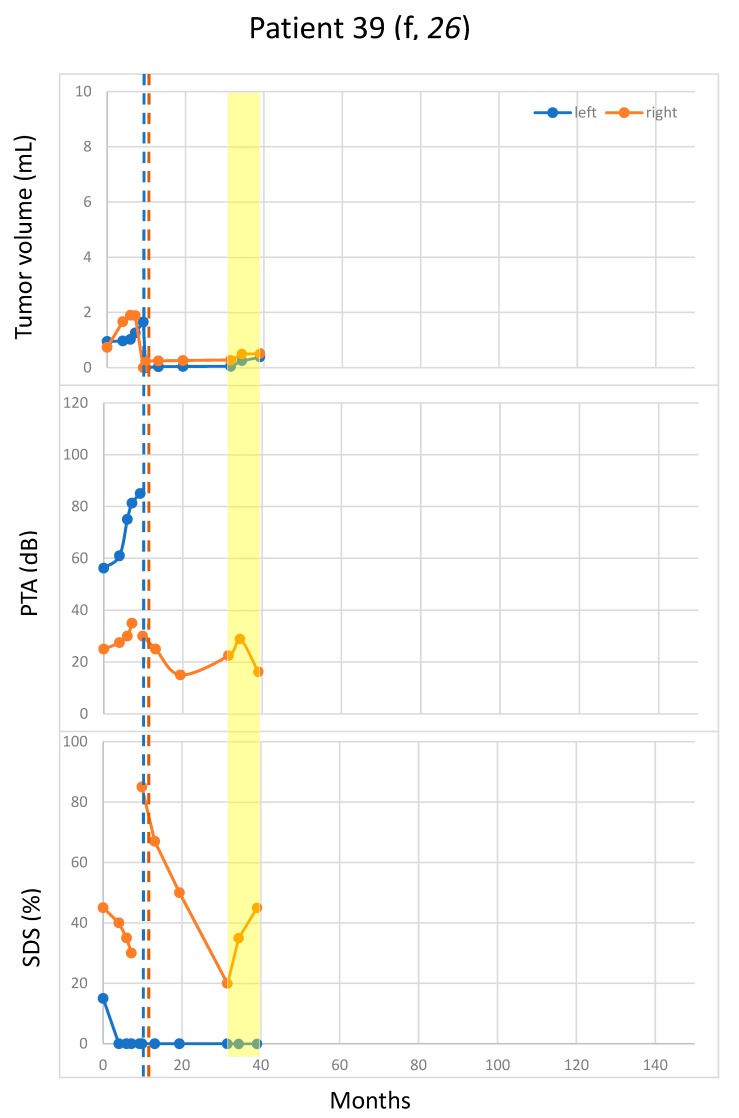

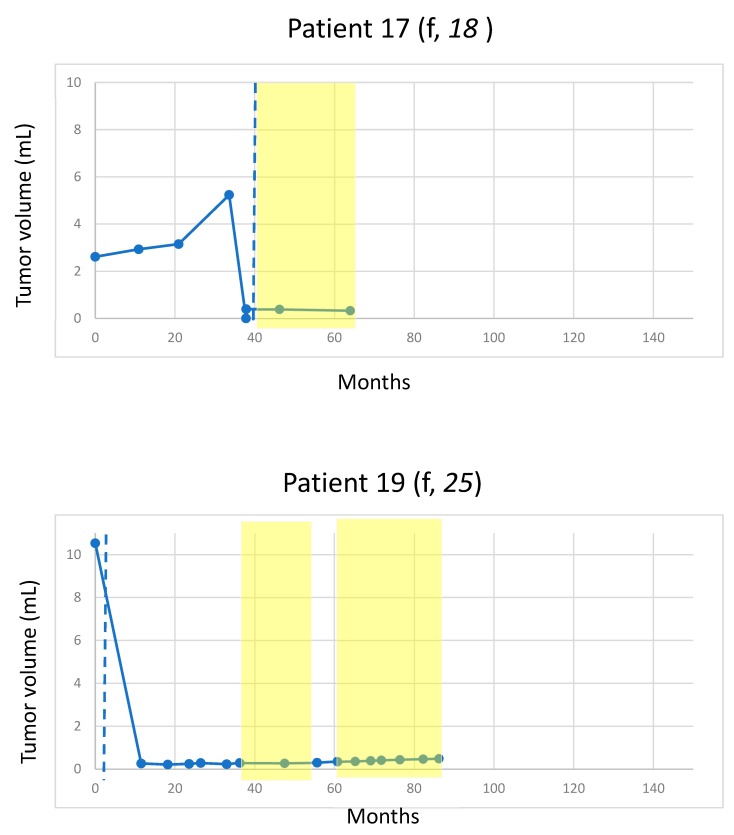
No or low growth of residual VS in both the treatment and the non-treatment periods. Tumor size was measured in volume (mL). Blue and orange dots represent the measurement for the left and right VS, respectively. Vertical blue and orange lines indicate the surgery times for the left and the right VS, respectively. Bevacizumab-treatment periods are marked in yellow. In all patients (Patients 10, 39, 17 and 19), significant volume reduction after surgery could be achieved. f: female; PTA: pure-tone average; SDS: speech discrimination score.

Pattern 4 (four patients): no or low growth in both the treatment and the non-treatment periods (Figure 4). 

For seven out of the nine patients, data were available for bilateral VS. Interestingly, in each of these patients, the response patterns of both VS were generally similar. Notably, in five patients (3, 5, 2, 10, 39), the growth curves of the bilateral VS were nearly parallel to each other. 

**Figure 5:**
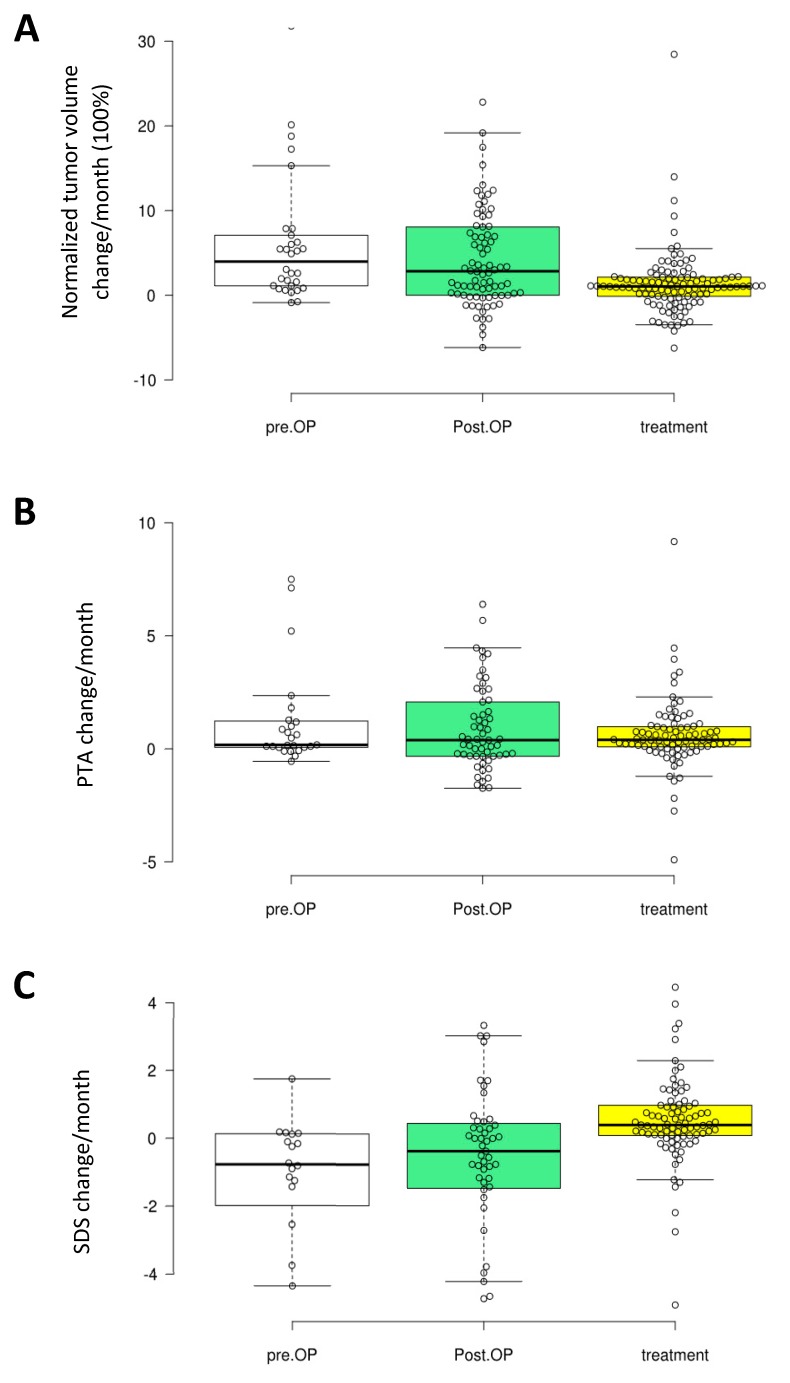
Box-plots showing change of tumor volume (**A**), changes of pure-tone average (PTA) (**B**) and of speech discrimination score (SDS) (**C**) between two measurement points in the treatment and the non-treatment periods, as well as in the period before surgery. For each parameter, the data sets in the three periods did not differ significantly from each other but a trend of slower growth and change of SDS in treatment periods was seen. pre.OP: preoperative; Post.OP: postoperative.

Growth of the residual VS in volume between two measurement points was slightly lower in the treatment period than in the non-treatment period. However, this difference was not significant (*p* = 0.19) and the variations were large (Figure 5A). 

### 2.4. Hearing

Rapid hearing deterioration in the non-treatment periods was observed in four patients: the right side of Patient 1 (Figure 1), the right side of Patients 11 and 3 (both in Figure 3), and the right side pure-tone average (PTA) of Patient 10 (Figure 4). However, rapid or continued hearing deterioration was also observed in some treatment periods: right-side speech discrimination score (SDS) of Patient 1 (Figure 1), right-side PTA and SDS of Patient 11 (Figure 2), and bilateral PTA and SDS of Patients 2 and 5 (Figure 3). 

In some cases, hearing deterioration correlated with tumor growth, with a typical example of both sides in Patient 2 (Figure 3). However, there were also cases where hearing deterioration did not straightforwardly correlate with tumor growth. For example, both the PTA and the SDS worsened rapidly for the 2nd off- and the 2nd on-periods, whereas the tumor remained rather stable (Figure 2). The right-side PTA of Patient 10 also had continuously deteriorated over a period of 40 months after the surgery despite absent tumor growth (volume < 0.1 mL). In this case, the SDS was lost at surgery (Figure 4). 

PTA increases between two measurement points were generally compatible in the treatment and the non-treatment periods (Figure 5B).

SDS decreases between two measurement points were slightly less in the treatment periods than in the non-treatment periods (Figure 5C). However, the difference was not significant (*p =* 0.13). The variation again was large for both the PTA and the SDS data. 

### 2.5. Influencing Factors on Tumor Growth Rate under Bevacizumab Treatment

Partial regression plots and a plot of studentized residuals against the predicted values detected linearity, and a Durbin–Watson statistic of 1.80 showed independence of residuals. Visual inspection of a plot of studentized residuals versus unstandardized predicted values detected homoscedasticity. No evidence of multicollinearity was seen, as assessed by tolerance values greater than 0.1. There were no studentized deleted residuals greater than ±3 standard deviations, no leverage values greater than 0.2, and no values for Cook′s distance above 1. The assumption of normality was met, as determined by a Q–Q Plot. 

The multiple regression model did not show significant influence of the factors (pre- and postoperative tumor volume, growth rate and resection amount) on the growth rate under treatment, *F*(4, 12) = 0.791, *p* = 0.553, adj. *R^2^* = 0.209. 

## 3. Discussion

Due to known adverse effects and potential long-term risks of bevacizumab, postponing this therapy as long as possible is a desirable approach, especially for young NF2 patients. We, therefore, investigated the effect of bevacizumab in a combined-treatment approach of primary hearing-preserving surgery [7] and subsequent bevacizumab treatment. 

The data of our present study showed very heterogenous results of the individual effects. In some patients, tumor growth rates slowed down, others responded partially and others not at all. In the group comparison, growth rates were decreased under bevacizumab but the significance level (*p* = 0.19) was not reached due to the high data scattering. 

A continuous hearing deterioration for both PTA and SDS values was observed in all cases, mostly with a rather rapid increased deterioration over time. Since bevacizumab treatment was started at a delayed time point, the overall hearing impairment was more advanced at the time of treatment start. In none of the cases were radiological regression of the tumor or improvement of hearing observed. 

Previously published series of bevacizumab treatment for vestibular schwannomas in NF2 patients achieved positive and promising results for both suppressing radiological tumor growth and for preserving/improving hearing [9,10,11]. However, those studies included mainly progressive and large tumors without surgery. By contrast, our study only included rather small residual tumors after partial resection with a postoperatively reduced growth rate, as we have shown in a preliminary study (Gugel et al. [6]). This difference may be the reason for the discrepancy in efficacy of bevacizumab treatment in this series. Interestingly, in a study by Morris et al. [12] and colleagues, they could show that despite large tumor volumes, younger patients not only have faster growth rates but also respond more poorly to bevacizumab treatment than older patients. Therefore, patients´ age may also be an important factor in the response to treatment. 

Genotype–phenotype correlations in NF2 patients are known and well investigated [4,5]. To date, its influence on the response of bevacizumab has not been investigated in detail. According to our data, patients with a worse or mixed response to bevacizumab (Pattern 2 and 3) seem to have a more severe genotype and phenotype (truncating mutations and high tumor loads). However, the number of patients is too low to draw conclusions from. In addition, the intensity of VEGF/VEGFR expression should be taken into account. A correlation towards tumor growth is known [13,14], and targeted studies to determine a possible medication response and dosage are necessary in times of individualized medicine. 

Long-term bevacizumab treatment is associated with various adverse effects. Particularly, cardiovascular and renal effects can occur in up to 50% of patients [9]. Larger cohorts and longer follow-up periods, especially in young patients, are still being awaited in order to learn the effects of long-term bevacizumab treatment. In addition, rebound of growth and hearing in drug-interrupting periods due to adverse events or indicated surgery should not be underestimated. As our data points also show, an acceleration in tumor growth is possible in the interrupted treatment (Figure 2), which could be critical for hearing and in the case of large tumors. 

Antihypertensive medication (e.g., ACE inhibitors) enable the side effects to be controlled or even stopped and thus, the bevacizumab application to be continued. Therefore, a certain benefit can be attributed in this respect. In contrast to other studies with metastatic colorectal cancer [15] or glioblastoma [16] patients, a relevant connection between the antihypertensive medication (e.g., angiotensin system inhibitors) and the response of bevacizumab with regard to tumor and hearing control could not be observed in our 2 patients. Not only the number of cases, but also the duration of application interval (Patient 17 approximately 3 months), were too low to draw relevant conclusions from this.

## 4. Material and Methods

### 4.1. Patients and Clinics

NF2 diagnosis was confirmed by clinical evaluation using the Baser diagnostic criteria for NF2 [17]. 9 NF2 patients under 25 years of age at the time of diagnosis were included in this retrospective analysis. Operations were performed on 16 tumors between 2004 and 2018 at the Department of Neurosurgery and Centre of Neurofibromatosis in Tübingen. The Ethics Board of the Medical Faculty and University Hospital of Tübingen approved this retrospective analysis (No 018/2019BO2). All operations were performed at our institution via the retrosigmoid approach by decompression of the internal auditory canal (IAC) with various resection amounts under continuous neurophysiological monitoring by two experienced neurosurgeons (Marcos Tatagiba, Martin Ulrich Schuhmann). In all patients, bevacizumab was offered as an off-label use in external institutions and hearing and tumor volume were monitored during the regular follow-ups in our department.

Indications for surgery as previously described [6] were: 1) large tumors (T4, Hannover Classification System [18]) on both sides with brainstem compression, and 2) continuing tumor growth and deterioration of brainstem auditory evoked potentials (BAEPs) and/or impairment of pure-tone audiometry and/or speech discrimination score (SDS).

Indications for bevacizumab treatment after surgery were: a) continued tumor growth, and/or b) further hearing deterioration of BAEP and/or impairment of PTA and/or SDS. For 8 out of 9 patients, mutation analysis was carried out as previously described [19]. One patient and/or their parents rejected mutation analysis. Adverse events and toxicity data were collected during follow-up visits and scored according to the Common Terminology Criteria for Adverse Events (CTCAE) version 5.0 (U.S. Department of Health & Human Services, National Institutes of Health, National Cancer Institute, Washington, CO, USA). In the treatment periods, patients received intravenous bevacizumab 5mg/kg every two weeks. 

### 4.2. Volumetry, Growth Rate and Hearing

Clinical and radiological follow-ups were routinely performed every 3 to 6 months. Tumor volumetry, growth rate and resection amount were performed, classified and calculated in 284 data sets as previously described [6]. Patients with hearing aids or implants (e.g., cochlear or auditory brainstem implant) were excluded. Hearing was assessed in all patients by regular determination of 4-frequency PTA, SDS and BAEP every three to six months after surgery. The mean follow-up after surgery was 36 months (range 13 to 63 months) without and 28 months (range 7 to 43 months) with bevacizumab. Hearing deterioration was calculated as an annual percentage deterioration compared to baseline values. 

### 4.3. Data Evaluation 

Tumor volume, PTA and SDS data were separated into 3 groups: (1) before surgery, (2) after surgery in the non-treatment periods and (3) after surgery and in the bevacizumab-treatment periods. Change of tumor volume, PTA and SDS between two measurement points were calculated as one value in the form of change/months. Since tumor size varies largely, the % change of tumor volume between the measurement points was normalized by dividing it with the tumor volume of the first one as:

100 × [volume 2 − volume 1]/[measurement 2 − measurement 1]/[volume 1].

These values were presented in box-plots. Values in the treatment and the non-treatment periods were compared using a t-test with two-tailed hypothesis and presumed equal variation. 

A multiple regression was run to estimate the influence of pre- and postoperative tumor volume, postoperative relative growth rate and resection amount on the relative tumor growth rate under bevacizumab treatment.

## 5. Conclusions

Secondary postoperative bevacizumab treatment in the case of further tumor growth and/or hearing deterioration can slow down tumor growth rate in some cases or prevent exponential tumor growth, but the effect on hearing seems negligible. However, the response is individual, heterogenous and may be dependent on influencing factors (e.g., genotype–phenotype and VEGF/VEGFR expression) and should be further investigated in larger cohorts. Treatment decision in NF2 patients remains individual. The risk of accelerated growth in periods of interruption or after termination of medication due to adverse events should be well considered and not underestimated. 

## Figures and Tables

**Table 1:** **Clinical** and genetic data of the nine neurofibromatosis type 2 (NF2) patients.

Patient	Sex	Age	*NF2* Mutation	Additional Tumor Load
10	f	29	No mutation found	Intraspinal extramedullary tumors
39	f	26	Nonsense in exon 6	Intracranial non-vestibular schwannomas and meningiomas, intraspinal extramedullary tumors
19	f	25	No mutation found (Mosaic)	Intracranial meningiomas and non-vestibular schwannomas, intraspinal intra- and extramedullary tumors
11	f	23	Frameshifting in exon 1	Intracranial meningiomas, intraspinal extramedullary tumors
2	f	21	Frameshifting in exon 5	Intracranial meningiomas, intraspinal extramedullary tumors
17	f	18	Not analysed	Intracranial meningiomas, intraspinal extramedullary tumors
1	m	21	No mutation found (Mosaic)	Intracranial meningiomas and non-vestibular schwannomas, intraspinal intra- and extramedullary tumors
5	m	19	Frameshifting in exon 5	Intracranial meningiomas and non-vestibular schwannomas, intraspinal extramedullary tumors
3	f	17	Nonsense in exon 6	Intracranial meningiomas and non-vestibular schwannomas

f: female; m: male.

**Table 2:** Demographic data of nine young NF2 patients (16 tumors).

Operation Side	
Left	9
Right	7
Age at diagnosis in years (mean ± std, range)	12 ± 7, 1–20
Age at surgery in years (mean ± std, range)	16 ± 5, 8–23
Age at beginning with BVZ in years (mean ± std, range)	19 ± 4, 14–26
Postoperative follow-up period without BVZ in months (mean ± std, range)	36 ± 26, 13–63
Postoperative follow-up period with BVZ in months (mean ± std, range)	28 ± 14, range 7–43
Tumor volume in cm^3^ (mean ± std, range)	
- preoperative	2.3 ± 2.8, 0.2–10.5
- postoperative native	0.9 ± 1.4, 0.03–5
- postoperative with BVZ	2.0 ± 1.8, 0.2–6.5
Resection amount	
- only bony decompression	1
- decompression with laser coagulation	1
- partial (<90%)	12
- subtotal (≥90–<95%)	2
- near total (≥95–<100%)	0
- total (100%, inclusive tumor capsule)	0

BVZ: bevacizumab; std: standard deviation.
